# Impact of the Smarter Safer Homes Solution on Quality of Life and Health Outcomes in Older People Living in Their Own Homes: Randomized Controlled Trial

**DOI:** 10.2196/59921

**Published:** 2025-01-22

**Authors:** Wei Lu, David Silvera-Tawil, Hwan-Jin Yoon, Liesel Higgins, Qing Zhang, Mohanraj Karunanithi, Julia Bomke, Joshua Byrnes, Jennifer Hewitt, Vanessa Smallbon, Jill Freyne, Deepa Prabhu, Marlien Varnfield

**Affiliations:** 1 Australian e-Health Research Centre Commonwealth Scientific and Industrial Research Organisation Brisbane, QLD Australia; 2 Research Center for Frontier Fundamental Studies Zhejiang Lab Hangzhou, ZheJiang China; 3 Child Health Research Centre The University of Queensland Brisbane, QLD Australia; 4 Centre for Applied Health Economics Griffith University Brisbane, QLD Australia; 5 Sydney School of Health Sciences Faculty of Medicine and Health The University of Sydney Sydney, NSW Australia

**Keywords:** randomized controlled trial, digital health, eHealth, smart home, sensor, health monitoring, home monitoring, aged care, aging in place, older adult, quality of life

## Abstract

**Background:**

An increasingly aging population, accompanied by a shortage of residential aged care homes and workforce and consumer feedback, has driven a growing interest in enabling older people to age in place through home-based care. In this context, smart home technologies for remote health monitoring have gained popularity for supporting older people to live in their own homes.

**Objective:**

This study aims to investigate the impact of smart home monitoring on multiple outcomes, including quality of life, activities of daily living, and depressive symptoms among older people living in their own homes over a 12-month period.

**Methods:**

We conducted an open-label, parallel-group randomized controlled trial. The control group continued to receive their existing care from aged care service providers. Meanwhile, the intervention group, in addition to receiving their usual aged care services, had their activities of daily living monitored using a smart home platform. Surveys including the Adult Social Care Outcomes Toolkit (ASCOT), EuroQol-5 Dimensions-5 Levels (EQ-5D-5L), Katz Index of Independence in Activities of Daily Living (Katz ADL), Lawton Instrumental Activities of Daily Living Scale (IADL), and Geriatric Depression Scale (GDS) were conducted at baseline and 6 and 12 months from baseline. Linear mixed-effects models were used to compare the difference between the intervention and control groups, with the ASCOT as the primary outcome measure.

**Results:**

Data from 130 participants were used in the analysis, with no significant differences in baseline characteristics between the control group (n=61) and the intervention group (n=69). In comparison to the control group, the intervention group had a higher ASCOT score at the 6-month assessment (mean difference 0.045, 95% CI 0.001 to 0.089; Cohen *d*=0.377). However, this difference did not persist at the 12-month assessment (mean difference 0.031, 95% CI –0.014 to 0.076; Cohen *d*=0.259). There were no significant differences in EQ-5D-5L, Katz ADL, IADL, and GDS observed between the intervention and control groups at the 6-month and 12-month assessments.

**Conclusions:**

The study demonstrates that smart home monitoring can improve social care–related quality of life for older people living in their own homes. However, the improvement was not sustained over the long term. The lack of statistically significant findings and diminished long-term improvements may be attributed to the influence of the COVID-19 pandemic during the later stage of the trial. Further research with a larger sample size is needed to evaluate the effect of smart home monitoring on broader quality-of-life measures.

**Trial Registration:**

Australian New Zealand Clinical Trials Registry ACTRN12618000829213; https://tinyurl.com/2n6a75em

**International Registered Report Identifier (IRRID):**

RR2-10.2196/31970

## Introduction

Globally, the aging population is steadily increasing, with 703 million individuals aged 65 years or over as of 2019, and this number is projected to reach 1.5 billion by the year 2050, with a global average life expectancy of around 77.1 years [1]. By 2041, the population of Australia aged 65 years and older is expected to see a 54% increase, rising from 4.31 million in 2021 to 6.66 million [2]. This demographic trend presents unique challenges in providing adequate care and support for older individuals, particularly those who choose to age in their own homes. Residential aged care facilities have traditionally been the primary setting for delivering aged care services. However, an aging population, coupled with a global shortage of aged care facilities and workforce, poses significant challenges to residential aged care. This leads to compromised access to essential care services, overburdened existing facilities, and increased strain on caregivers, impacting the quality of care [3-5]. To address these challenges, there has been a growing policy emphasis on aging in place, the concept of older people living longer in their own homes and communities as they age [6,7]. This allows older people to remain connected to their communities, friends, and family networks, promoting overall well-being and social support. Aside from older people’s preference, the lack of residential care placements and the rising costs associated with institutional care make aging in place a more feasible and cost-effective option for many individuals.

In this context, smart home technologies have emerged as a promising solution to support the well-being and independence of older individuals living in their own homes. These technologies encompass a wide range of devices and systems, including sensor networks, remote monitoring, and assistive devices, which can enhance safety, health monitoring, and overall quality of life for older adults [8,9]. By integrating these technologies into the home environment, smart home solutions have the potential to address various challenges associated with aging in place, such as falls, medication adherence, and social isolation [10].

While the potential benefits of smart home solutions in supporting aging in place have been widely acknowledged, the evidence base, particularly from randomized controlled trials (RCTs), remains limited [11,12]. The evaluation of smart home technologies through RCTs is important to establish their efficacy, effectiveness, and cost-effectiveness in real-world settings. Moreover, previous studies investigating the influence of smart home technologies on the well-being of older individuals largely focused on health-related quality of life [13-16]. Smart home technologies can detect emergencies, monitor health, and assist in daily tasks, which can lead to increased autonomy and security, not only promoting physical well-being but also addressing critical social and emotional needs [11,17]. However, an increased emphasis on health-related quality of life and less attention to the social care–related quality of life has created a gap in our understanding of how smart home technologies can affect broader aspects of older people’s quality of life, beyond health functioning [18-20].

Following our previously published protocol [21], we conducted an RCT, with the primary aim of evaluating how the use of the Smarter Safer Home (SSH) platform impacts the social care–related quality of life in older people living in their own homes. As a secondary aim, we examined whether this technology impacts the health-related quality of life, ability to perform activities of daily living (ADLs), and severity of depression. The findings of this study enhance our understanding of how smart home technologies can impact older people who live in their own homes. Moreover, they have the potential to inform policy and practice in the field of aged care, providing valuable insights into the effectiveness and feasibility of implementing smart home solutions to support aging in place.

## Methods

### Study Design

This study is a mixed-methods, open-label, parallel-group RCT. Participants were allocated randomly to a control group and an intervention group. The control group received regular aged care services subsidized by the Australian government, which can be categorized into 2 types: the Commonwealth Home Support Programme (CHSP) and the Home Care Package (HCP). CHSP provides basic support to help older Australians live independently, while HCP offers more comprehensive and personalized care for those with higher or more complex needs. The intervention group, in addition to usual aged care services, had the SSH platform installed at their residence. The SSH platform is an in-home monitoring system that uses distributed ambient sensors to track the physical environment and human activities within a household. The sensor data is transmitted to a cloud computing server that assesses residents' ADLs and sleep patterns. During the trial, the assessment was accessible to participants, their families, and aged care service providers through a tablet app or web portals. Survey questionnaires completed by the participants were used to measure primary and secondary outcomes at baseline, 6 months, and 12 months. Additionally, the trial collected various other types of data, including raw sensor data, evaluations from both informal and formal carers, participants' health services utilization, and log spreadsheets kept by aged care service providers detailing their usage of the SSH system [21]. This paper is specifically focused on the survey questionnaires completed by the participants. Analyses of the additional data types collected during this RCT will be presented in separate publications.

### Ethical Considerations

Ethics approval for this study was obtained from the Commonwealth Scientific and Industrial Research Organisation (CSIRO) Human and Medical Research Ethics Committee (CHMREC; HREC 4/2018). The trial was registered at the Australian New Zealand Clinical Trials Registry (ACTRN12618000829213). All participants consented to join the study. They are free to withdraw or suspend their participation at any time with no need to provide a reason.

### Inclusion and Exclusion Criteria

The inclusion criteria were as follows: (1) older adults (65 years and older), (2) living at home, in the care of a designated aged care service provider, and (3) English-speaking with proficiency in written English. The exclusion criteria were as follows: (1) residing in long-term residential care, (2) unable to give informed consent due to reasons such as severe cognitive impairment, and (3) unwilling to leave their electricity on overnight.

### SSH Platform

**Figure 1:**
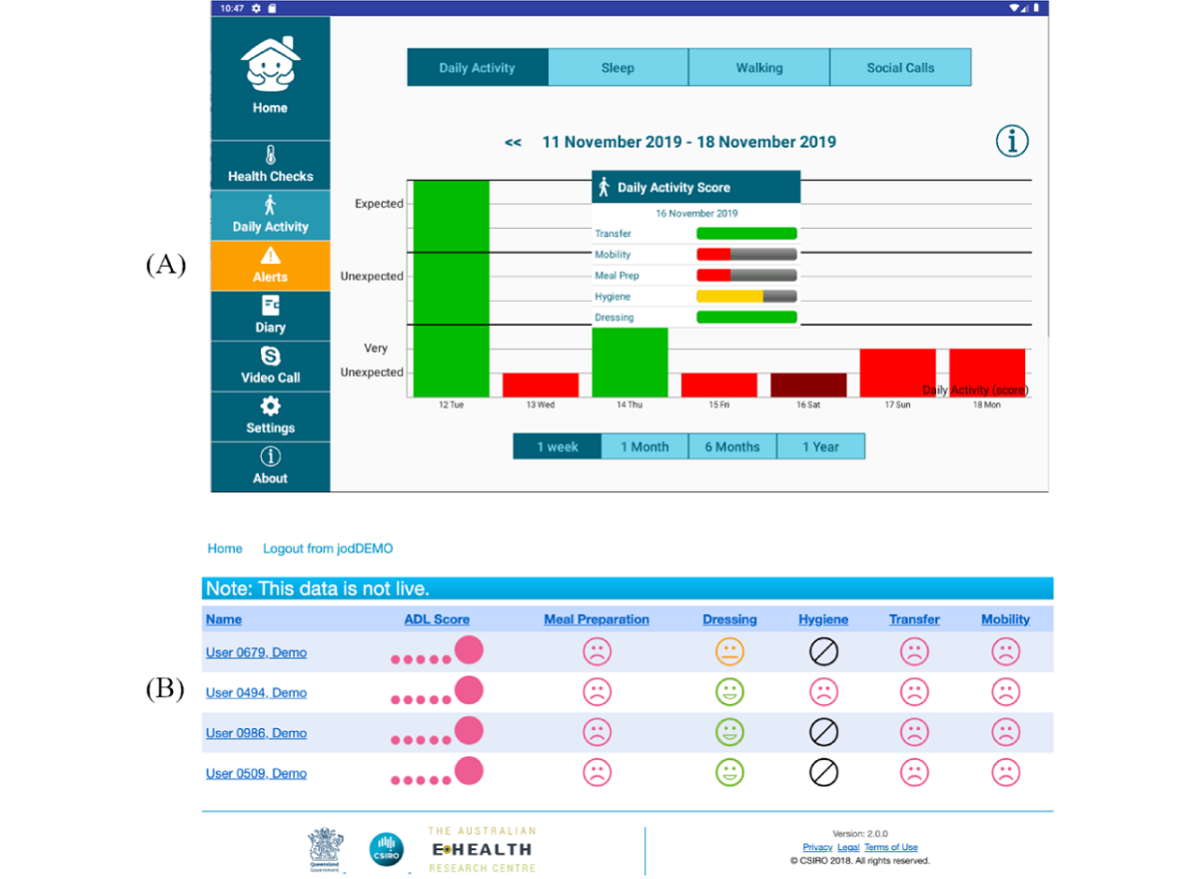
The user interface of (A) the SSH tablet app and (B) the SSH web portal. SSH: Smarter Safer Home.

This study used the SSH platform presented in the protocol paper [21]. The SSH platform included an in-home network of distributed sensors, a cloud server, and a client module with a tablet app, a family web portal, and a service provider web portal (Figure 1). The sensor network consisted of ambient sensors that measured the physical environment (eg, room temperature, humidity) as well as human activities (eg, motion, door opening or closing) within the home. The sensors wirelessly sent data to a gateway hub, which relayed the data to a secure cloud server through a local WiFi network. A cloud computing service was implemented to process the sensor data, assessing the resident’s ADLs across 5 domains, including mobility, transfer, hygiene, dressing, and meal preparation. The ADL scores were then compared with the participant’s individual baseline profile established with the data collected over the initial 21 days of the trial period. Deviations from the baseline in each ADL domain were categorized as expected, unexpected, or very unexpected. Apart from ADL performance, the resident’s sleep measures were also collected with sleep sensors placed under the bed mattress. The analytic results were made accessible to the participants, their families, and associated aged care service providers through a tablet app, a family web portal, and a service provider web portal, respectively.

The setting was home-based, with participants living in their own residences in the community. Participants were recruited from the customer bases of the aged care service providers in metropolitan and regional areas in Queensland, Australia, as defined by the Rural Remote Area and Metropolitan Area classification criteria [22].

### Trial Setting

The RCT was conducted between April 2019 and November 2020, in partnership with 3 aged care service providers (Textbox 1).

**Overview of aged care service providers involved in the study.**

- Anglicare Southern Queensland is a member of the Anglicare Australia Network providing support to aged Australians in partnership with government and other support organizations, in response to identified care needs throughout South East Queensland.
- integratedLiving Australia receives funding from the Australian and state governments to provide care services. They provide in-home support services to older people in regional, rural, and remote Australia (including North and Eastern Queensland) and have been focusing on ensuring equitable access for health support to these communities.
- All About Living partners with federal, state, and local government departments and a number of community organizations to deliver a range of high-quality services. All About Living has developed governance, management, and service delivery expertise and excellence that enable consistent service delivery.

### Recruitment

The aged care service providers identified eligible clients from their services and provided details to a team of project officers independent from the aged care service providers, who recruited and consented participants to the trial. The project officers contacted potential participants after an introduction by the aged care service provider. The project officer, via phone call, briefed the individual on the trial and assessed their interest in participating. If the individual agreed to participate, they were asked to nominate their informal carer to act as a witness in a future face-to-face meeting, where the project officer confirmed that the prospective participants met the inclusion criteria. Potential participants were provided with the Participant Information and Consent Form and a verbal description of the trial requirements by the project officer. Individuals who agreed to participate were asked to sign the Participant Information and Consent Form.

### Procedures

Once participants had been consented, baseline surveys were administered to them by the project officer. After completing the baseline surveys, participants were allocated randomly to either the intervention or control groups. Stratified randomization was used with metropolitan and regional strata to preserve the ratio of the 2 geographical areas in both intervention and control groups. An independent researcher conducted the randomization using a computer program and anonymized identifiers. All participants (intervention and control) continued to receive their existing care and social services in line with local aged care service provider protocols for the 12 months of the trial. In addition, participants allocated to the intervention group received an SSH kit, including sensors, a tablet, and a gateway hub. The installation of the SSH kit was coordinated by the project officer, typically within 2 weeks of group allocation. All participants were contacted by a project officer to complete the follow-up surveys at 6 months and 12 months from baseline. The surveys were conducted either over the phone or in person and were managed using the REDCap (Vanderbilt University) hosted by CSIRO. During the trial period, the project officer was the point of contact for any problems or questions. If an intervention group participant wished to withdraw from the trial, the project officer arranged for the SSH kit to be uninstalled from their home as quickly as possible. Upon completion of the project, all homes had their SSH kits removed by the project officer.

### Intervention

The intervention involved monitoring ADLs of participants through the SSH platform. During the trial, the aged care service providers aimed to review the trends in the participants’ ADL performance through the SSH service provider portal 5 days a week, specifically on business days. When the aged care service providers identified a participant’s ADL performance to be in an unexpected or very unexpected range for 2 consecutive days, they would contact the participant via phone or send an email to the participant’s case manager. The aged care service providers created their own intervention logs to record information about actions taken for the participants. Given the workload on aged care service providers, maintaining the intervention log was not a compulsory task to form a complete record of the interventions. However, it offered some insights into how aged care service providers used the SSH platform to trigger interventions. Although no specific tasks were mandated, participants and their families were encouraged to monitor the participants’ ADL performance and take any actions they deemed appropriate.

### Outcome Measures

The primary outcome measure was social care–related quality of life, measured with a self-completion version of the Adult Social Care Outcomes Toolkit (ASCOT [23]). Secondary outcomes included EuroQol-5 Dimensions-5 Levels (EQ-5D-5L [24,25]), Katz Index of Independence in Activities of Daily Living (Katz ADL [26]), Lawton Instrumental Activities of Daily Living Scale (IADL [27]), and Geriatric Depression Scale (GDS [28]).

### Sample Size Calculation

The power calculation for the randomized trial design was based on a clinically significant difference of 10% in ASCOT, with a distribution of 0.80 (SD 0.16) as indicated in prior research [29]. Given a null hypothesis of α≥.05, an effect size of 0.5 (0.08/0.16), identical allocation ratio for the 2 groups in our trial, and a 30% attrition rate, the sample size of this trial was computed to be 100 participants per group to achieve 80% statistical power [21].

### Statistical Analysis

Descriptive statistics summarized the baseline characteristics and outcome measures by groups. For continuous variables and categorical variables, means and SD or numbers and percentages were reported, respectively. The primary and secondary outcome measures were analyzed using a linear mixed-effects model to compare the difference between the intervention and control groups. Fixed effects included group allocation, time (6 months and 12 months from baseline), group-by-time interaction, and baseline scores. Additionally, the analysis considered Participant ID as a random effect to account for within-individual correlations. Cohen *d* was used to describe the standardized mean difference of an effect. We conducted sensitivity analyses to examine whether the impact of the SSH platform varied across subgroups defined by baseline demographics, such as gender, care package, and living status. Marriage status and aged care service provider were not included in the sensitivity analyses due to insufficient sample sizes in certain subcategories.

### Data Exclusion

#### COVID-19 Impact

In 2020, while the trial was ongoing, Australia was struck by the COVID-19 pandemic, presenting the country's older community with unexpected and significant challenges. Older adults faced considerable health risks due to their vulnerability to severe illness from COVID-19. Additionally, public health restrictions such as lockdowns and social distancing imposed by the Australian government could have both direct and indirect effects on the physical and mental health of older adults [30-32]. While quantifying the exact impact of the pandemic is challenging, we performed data exclusion to ensure consistency in the pandemic’s impact on outcome measures within the 6-month and 12-month assessments.

There were 2 distinct peaks in COVID-19 cases in Australia during the trial period. The first wave, characterized by infections imported from overseas, peaked in March and April 2020. The second wave, largely resulting from increased community transmission, peaked in August 2020 and subsided by October 2020 [33]. Therefore, we identified 2 key dates, April 26, 2020, and October 26, 2020, marking the progression of COVID-19 transmission and the implementation of Australian government public health measures.

April 26, 2020: The Queensland government applied mandatory social distancing, following a series of measures, including restrictions on home confinement, movement, and gathering [34].

October 26, 2020: The end of Australia’s second wave of COVID-19 infections in 2020 [35,36].

During the period from April 26 to October 26, 2020, the participants presumably experienced a heightened impact of COVID-19 due to more strict government measures and increased local transmission compared to periods outside this timeframe. Notably, most of the 6-month surveys were administered prior to April 26, 2020, while most 12-month surveys were conducted between April 26 and October 26, 2020. Taking these factors into account, we excluded 6-month surveys conducted after April 26, 2020, and 12-month surveys conducted either before April 26, 2020, or after October 26, 2020. As a result, the 6-month and 12-month assessments were separated into different scenarios, reflecting periods of lower and higher COVID-19 impact, respectively.

#### Temporal Precision

The 6-month and 12-month surveys were designed to assess participants’ outcome measures at 6 months and 12 months from the baseline, respectively. However, the actual timing of these surveys varied depending on participant availability. To enhance the temporal precision of the survey results, we excluded data collected outside a 1-month window around the intended survey time.

## Results

### Participants

**Figure 2:**
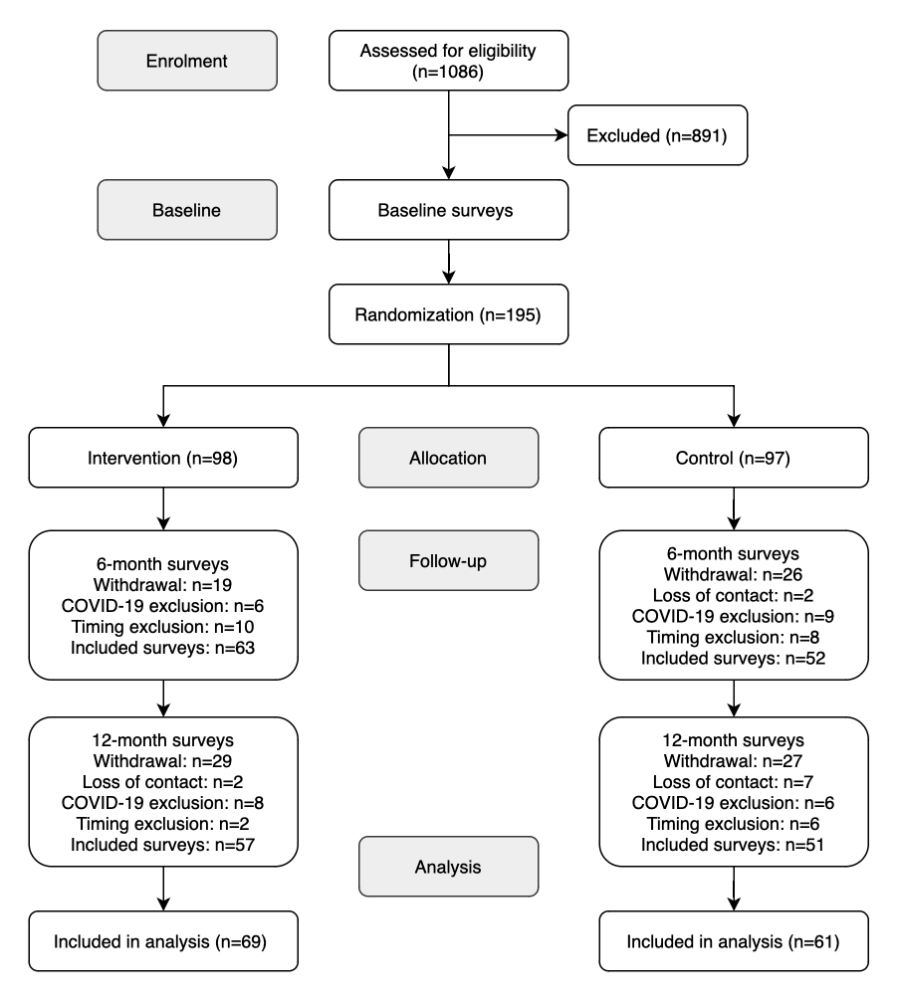
Flow diagram of inclusion process and number of included participants.

A total of 1086 clients of our partnering aged care service providers were assessed. From those, 195 provided consent to participate in the trial and completed the baseline surveys (Figure 2). Randomization resulted in 97 participants in the control group and 98 participants in the intervention group. The final analysis included the surveys from 61 participants in the control group and 69 participants in the intervention group. The absence of follow-up surveys can be attributed to participants' withdrawals (n=56) or loss of contact (n=9). Additionally, 29 surveys were excluded to account for the impact of COVID-19, and 26 surveys were excluded to ensure the temporal precision of the data.

In total, across the 73 participants included in the aged care service provider’s intervention logs, on 284 occasions a decision was made to contact the participant. The contact was made either directly with the participant (144 instances) or through an email sent to the participant’s case manager, requesting they contact the participant (140 instances).

### Baseline Characteristics

The sample included 130 participants, with 69 in the intervention group and 61 in the control group. The average age of all participants at the baseline was 82.4 (SD 7.3) years.

Most participants were male (70% overall), with a slightly higher percentage of males in the control group (73.8%) compared to the intervention group (66.7%). Both groups received their regular aged care services, either CHSP (45.4%) or HCP (54.6%). In terms of baseline survey results, there were slight variations between the intervention and control groups in ASCOT, EQ-5D-5L, Katz ADL, IADL, and GDS scores. Additional details are available in Table 1.

**Table 1:** Baseline characteristics.

Characteristics		Overall (n=130)	Intervention (n=69)	Control (n=61)
**Age (years), mean (SD**)		82.4 (7.3)	82.7 (7.0)	82.1 (7.7)
**Gender, n (%)**				
	Female	39 (30)	23 (33.3)	16 (26.2)
	Male	91 (70)	46 (66.7)	45 (73.8)
**Care package, n (%)**				
	CHSP^a^	59 (45.4)	31 (44.9)	28 (45.9)
	HCP^b^	71 (54.6)	38 (55.1)	33 (54.1)
**Marriage status, n (%)**				
	Not married	6 (4.7)	3 (4.3)	3 (4.9)
	Married	49 (38)	26 (37.7)	23 (37.7)
	Widowed	57 (44.2)	31 (44.9)	26 (42.6)
	Divorced	13 (10.1)	7 (10.1)	6 (9.8)
	Separated	5 (3.1)	2 (2.9)	3 (4.9)
**Living status, n (%)**				
	Alone	72 (55.4)	35 (50.7)	37 (60.7)
	With other	58 (44.6)	34 (49.3)	24 (39.3)
**Aged care service provider, n (%)**				
	Anglicare Southern Queensland	85 (65.4)	45 (65.2)	40 (65.6)
	integratedLiving Australia	28 (21.5)	12 (17.4)	16 (26.2)
	All About Living	17 (13)	12 (17.4)	5 (8.2)
**Baseline surveys, mean (SD)**				
	ASCOT^c^	0.859 (0.147)	0.866 (0.148)	0.852 (0.148)
	EQ-5D-5L^d^	0.724 (0.258)	0.726 (0.244)	0.721 (0.274)
	Katz ADL^e^	5.256 (1.201)	5.412 (0.918)	5.082 (1.441)
	IADL^f^	4.969 (2.162)	4.910 (2.058)	5.033 (2.287)
	GDS^g^	3.146 (2.512)	3.319 (2.518)	2.951 (2.513)

^a^CHSP: Commonwealth Home Support Programme.

^b^HCP: Home Care Package.

^c^ASCOT: Adult Social Care Outcomes Toolkit ranges from –0.17 to 1, with higher scores indicating better social care–related quality of life.

^d^EQ-5D-5L: EuroQol-5 Dimensions-5 Levels ranges from –0.301 to 1, with higher scores indicating better health-related quality of life.

^e^Katz ADL: Katz Index of Independence in Activities of Daily Living ranges from 0 to 6, with higher scores indicating greater independence in daily activities.

^f^IADL: Lawton Instrumental Activities of Daily Living Scale ranges from 0 to 8, with higher scores indicating greater ability to perform instrumental activities of daily living.

^g^GDS: Geriatric Depression Scale ranges from 0 to 15, with higher scores indicating more severe depression symptoms.

### Primary and Secondary Outcomes

Table 2 presents the effects of the SSH platform for the intervention and control groups on primary and secondary outcomes. At 6 months, there was a statistically significant difference (*P*=.046) in ASCOT scores between the intervention and control groups. The mean difference indicated that the intervention group had a 0.045 (95% CI 0.001 to 0.089) higher ASCOT score than the control group. The Cohen *d* effect size of 0.377 indicated a moderate effect in favor of the intervention group. At 12 months, the mean difference between the intervention and the control group reduced to 0.031 (95% CI –0.014 to 0.076; *P*=.18). The Cohen *d* effect size of 0.259 also suggested a smaller effect size compared to the 6-month assessment. The overall test of the group variable suggests there was a statistically significant difference in ASCOT scores between the intervention and control groups (*P*=.04). However, the overall test of group × time interaction was not significant (*P*=.59).

As shown in Table 2, there were no statistically significant differences between the 2 groups in the secondary outcomes (EQ-5D-5L, Katz ADL, IADL, and GDS) at 6 months or 12 months.

**Table 2:** Effects of the Smarter Safer Home platform for the intervention and control groups on primary and secondary outcomes.

Outcome					Intervention, mean (SD)		Control, mean (SD)		Mean difference (95% CI)		*P* value		Cohen *d* (95% CI)		Group, *P* value			Group × time, *P* value		
**ASCOT^a^**																.04				.59
	6 months				0.882 (0.119)		0.837 (0.118)		0.045 (0.001 to 0.089)		.046		0.377 (0.008 to 0.749)							
	12 months				0.855 (0.118)		0.824 (0.118)		0.031 (–0.014 to 0.076)		.18		0.259 (–0.119 to 0.640)							
**EQ-5D-5L^b^**																.15				.26
			6 months	0.745 (0.217)		0.669 (0.216)		0.076 (–0.004, 0.157)		.06		0.349 (–0.019 to 0.721)								
			12 months	0.654 (0.216)		0.634 (0.216)		0.020 (–0.063, 0.102)		.64		0.090 (–0.288 to 0.468)								
**Katz ADL^c^**																.65				.90
			6 months	5.123 (0.992)		5.038 (0.986)		0.085 (–0.284 to 0.454)		.65		0.085 (–0.282 to 0.453)								
			12 months	4.930 (0.984)		4.875 (0.985)		0.054 (–0.325 to 0.434)		.78		0.055 (–0.325 to 0.435)								
**IADL^d^**																.70				.10
		6 months			4.623 (1.046)		4.498 (1.043)		0.125 (–0.264 to 0.514)		.53		0.119 (–0.248 to 0.487)							
		12 months			4.357 (1.043)		4.610 (1.040)		–0.253 (–0.652 to 0.146)		.21		–0.241 (–0.622 to 0.137)							
**GDS^e^**																.26				.21
		6 months			3.479 (1.976)		3.575 (0.273)		–0.096 (–0.831 to 0.639)		.80		–0.048 (–0.416 to 0.319)							
		12 months			3.673 (1.958)		4.289 (0.275)		–0.617 (–1.368 to 0.134)		.11		–0.312 (–0.694 to 0.066)							

^a^ASCOT: Adult Social Care Outcomes Toolkit ranges from –0.17 to 1, with higher scores indicating better social care–related quality of life.

^b^EQ-5D-5L: EuroQol-5 Dimensions-5 Levels ranges from –0.301 to 1, with higher scores indicating better health-related quality of life.

^c^Katz ADL: Katz Index of Independence in Activities of Daily Living ranges from 0 to 6, with higher scores indicating greater independence in daily activities.

^d^IADL: Lawton Instrumental Activities of Daily Living Scale ranges from 0 to 8, with higher scores indicating greater ability to perform instrumental activities of daily living.

^e^GDS: Geriatric Depression Scale ranges from 0 to 15, with higher scores indicating more severe depression symptoms.

### Sensitivity Analyses

Significant improvements in ASCOT scores were observed, particularly among female participants (mean difference 0.089, 95% CI 0.016 to 0.162; *P*=.02) and HCP recipients (mean difference 0.079, 95% CI 0.015 to 0.142; *P*=.02) at 6 months (Tables S1 and S2 in Multimedia Appendix 1). Participants living with others showed a marginal improvement in ASCOT (mean difference 0.057, 95% CI –0.003 to 0.117; *P*=.06) at 6 months (Table S3 in Multimedia Appendix 1). The impact of the SSH platform on ASCOT for male participants, CHSP recipients, and participants living alone was not statistically significant. The impact on EQ-5D-5L, Katz ADL, IADL, and GDS was generally consistent with the main analysis (Tables S1, S2, and S3 in Multimedia Appendix 1).

## Discussion

### Principal Findings

In this RCT, 195 older people living in place were randomly assigned to intervention (using the SSH platform) and control groups. The analysis included 130 participants following withdrawals, loss of contact, and data exclusion. The effects of the SSH platform were assessed through the ASCOT, EQ-5D-5L, Katz ADL, IADL, and GDS collected at baseline, 6 months, and 12 months. The results demonstrated significant improvements in the primary outcome, social care–related quality of life, with the intervention group displaying higher ASCOT scores compared to the control group at 6 months. However, the long-term effect of the smart home solution on social care–related quality of life, as observed in the 12-month assessment, did not align with the initial findings.

The improvement in ASCOT can be interpreted through several factors related to the characteristics of the SSH platform. ASCOT measures different aspects of quality of life that can be affected by social care, such as safety, social participation, and dignity. The SSH platform’s features, including daily updates on activities and carer involvement, can improve social care–related quality of life by providing a sense of security and connectivity. On the other hand, EQ-5D-5L, Katz ADL, and IADL are associated with functional capacities and daily activities that may require specific interventions that the SSH platform does not directly address. For GDS scores, while the SSH platform can provide a sense of security and support, it may not be sufficient to significantly impact depressive symptoms without targeted interventions.

The diminished impact of the SSH platform on ASCOT observed at 12 months might be explained by an unprecedented peak of local COVID-19 transmission and strict public health measures. This is consistent with the studies indicating that the COVID-19 pandemic, characterized by concerns about virus transmission, limitations on social interactions, and disruption of daily activities, can lead to increased loneliness and reduced quality of life [37,38]. Therefore, the ability of smart home solutions to enhance the social care–related quality of life among older individuals needs continued adaptation to external factors, such as the COVID-19 pandemic, which can influence these outcomes.

Although the mean difference in secondary outcomes was not statistically significant, the intervention group demonstrated better point estimates (higher scores in EQ-5D-5L, Katz ADL, and IADL, and lower scores in GDS) compared to the control group at 6 months. Additionally, the estimates of mean difference in the GDS scores showed an increase by the 12-month assessment. However, this study’s sample size might have limited the power to detect the effect of the SSH on the secondary outcomes.

### Limitations

When interpreting the results of this study, some limitations must be taken into account. The primary limitation stemmed from the unforeseen impact of COVID-19 on trial operations and data analysis. First, implementing blinding for group allocation in this trial was not feasible due to the nature of the intervention, which required installation of SSH in participants’ houses. The COVID-19 pandemic had adverse effects on the mental and physical health of older individuals, leading to increased rates of depression, anxiety, and cognitive decline, ultimately affecting their overall quality of life [30-32]. We excluded some data to maintain the consistency of COVID-19’s impact on outcome measures at the same time points. This effectively separated the 6-month assessment and 12-month assessment into different scenarios with lower and higher COVID-19 impact, respectively. The adverse impact of COVID-19 might have, to some degree, overshadowed the effects of the SSH platform during the 12-month assessment, influenced by the heightened transmission of COVID-19 and the implementation of public health measures in Australia. In addition, the reduction in sample size from the initial sample size calculation may have reduced the statistical power of the study to detect a difference between groups.

While this study aimed to investigate how the SSH impacts older adults living in their own homes, the original protocol [21] excluded those residing with others, placing an additional emphasis on independent living. However, during the initial phase of recruitment, it became apparent that excluding older adults living with others significantly limited the number of participants available for recruitment and compromised the final sample size. Consequently, we removed this exclusion criterion. This adjustment allowed us to enhance our recruitment efforts and more effectively pursue the main objective of this study.

In addition, among the 195 participants recruited in this trial, 47 participants did not complete the 6-month assessment, and 65 participants did not complete the 12-month assessment. We noted that the participants without 6-month or 12-month follow-up data had lower EQ-5D-5L or IADL scores at baseline (Table S4 in Multimedia Appendix 1).

### Comparison With Prior Work

Quality of life has been extensively investigated as one of the health outcomes associated with the use of smart home technologies [9,12,39,40]. However, most studies have primarily examined the impact of these technologies on health-related quality of life, with limited focus on social care–related quality of life within the smart home context. An RCT found that telemonitoring of biometric data along with administering symptom questionnaires did not significantly improve the mental health component of health-related quality of life. However, it may have led to a worsened physical component of health-related quality of life in the telemonitored group compared to the usual care group over the study period [16]. In another RCT of an in-home weight monitoring system in patients with advanced heart failure, the intervention group displayed an increasing trend in quality of life, but the difference was not statistically significant [15]. A systematic review reported that there was no evidence supporting that smart home and home health monitoring technologies improved the health-related quality of life for older adults [9]. Similarly, a meta-analysis of 5 RCTs investigating the effects of smart homes on older patients with chronic conditions indicated that the telemonitoring group did not yield a statistically significant impact on quality of life [12]. A scoping review examining the effectiveness of smart home technologies in supporting community-dwelling older adults with dementia found no significant effects on health-related quality of life in the 5 included studies [40]. In sum, the existing body of literature on RCTs related to smart home technologies has not conclusively demonstrated their positive influence on health-related quality of life. Moreover, evidence regarding the impact of smart home solutions on social care–related quality of life remains considerably limited.

In this study, a significant improvement in social care–related quality of life has been observed among the intervention group as compared to the control group after the first 6 months of using the SSH platform. Despite the heightened COVID-19 impact on the 12-month assessment, the intervention group displayed a possible improvement (mean difference 0.031, 95% CI –0.014 to 0.076) in social care–related quality of life at 12 months. The ASCOT employed in this study has been proven to be a promising measure to evaluate care services for older people by broadening the evaluative space beyond the health domain to well-being [41,42]. Several studies have recommended the use of the ASCOT in combination with the widely adopted EuroQol-5D (EQ-5D) to measure broader aspects of quality of life beyond physical functioning and health dimensions [18-20]. Even though statistical significance was not achieved in secondary outcome measures, including health-related quality of life, this study filled in a gap in existing literature by assessing the effect of smart home technologies on social care–related quality in community-dwelling older people. This study presented, for the first time, evidence from an RCT, highlighting the possibility of smart home technologies in enhancing the social care–related quality of life for older people living in their own homes.

### Future Directions

This RCT has yielded an extensive collection of data, including survey responses from older adult participants, data collected from smart home sensors, and evaluations from both informal and formal caregivers. Beyond this paper, there are several ongoing or planned future research projects that will use various data sources derived from this RCT. For instance, in an exploratory study, we analyzed the correlation between GDS and motion sensor data from participants' homes, aiming to determine if signs of depression could be detected through observed changes in in-home movement patterns [43]. This approach is currently being expanded through machine learning techniques to discover digital biomarkers for various health risks. In addition, a cost-utility analysis will be carried out to evaluate the cost-effectiveness of the intervention. Future research will also include a qualitative analysis to integrate the log spreadsheet maintained by aged care service providers, documenting the usage of SSH throughout the trial, with feedback from both participants and caregivers. The analysis of this data has the potential to open new avenues for predictive health analytics and proactive healthcare interventions. Besides, additional trials should be conducted to further evaluate the effect of the SSH platform on the secondary outcomes or in specific subgroups.

### Conclusions

This RCT is a pioneering step in the direction of assessing the impact of smart home technology on older adults’ well-being while living in their own homes. It uniquely focused on social care–related quality of life using the ASCOT, along with EQ-5D-5L, Katz ADL, IADL, and GDS measures. The study found significant improvements in ASCOT among participants using the SSH platform during the first 6 months. However, after 12 months, the improvement did not maintain statistical significance. The increased impact of the COVID-19 pandemic on the 12-month assessment was a noteworthy factor, as the pandemic introduced unforeseen challenges and restrictions that might have obfuscated the long-term effects of the SSH platform. While no significant changes were observed in the secondary outcome measures, this study contributes to understanding the potential of smart home technologies in enhancing social care–related quality of life, underscoring the need for further research to investigate the long-term effects and broader implications of such technologies in the care of older adults.

## Appendix

Multimedia Appendix 1 Sensitivity analyses and baseline characteristics of the participants with and without follow-up data.

Multimedia Appendix 2 CONSORT-eHEALTH checklist (V 1.6.1).

## Abbreviations

ADL: activities of daily living
ASCOT: Adult Social Care Outcomes Toolkit
CHSP: Commonwealth Home Support Programme
CSIRO: Commonwealth Scientific and Industrial Research Organisation
EQ-5D: EuroQol-5 Dimensions
EQ-5D-5L: EuroQol-5 Dimensions-5 Levels
GDS: Geriatric Depression Scale
HCP: Home Care Package
IADL: Lawton Instrumental Activities of Daily Living Scale
Katz ADL: Katz Index of Independence in Activities of Daily Living
RCT: randomized controlled trial
SSH: Smarter Safer Home
