# Supplementary material for: Impact of the Smarter Safer Homes Solution on Quality of Life and Health Outcomes in Older People Living in Their Own Homes: Randomized Controlled Trial
Source: J Med Internet Res. 2025 Jan 22;27:e59921. doi: 10.2196/59921 (PMC11799804; doi:10.2196/59921)
Supplement: Multimedia Appendix 1 [file jmir_v27i1e59921_app1.pdf]

Table S1 Sensitivity analysis for subgroups defined by gender

	Female				Male			
	Intervention, mean (SD)	Control, mean (SD)	Mean difference, (95% CI)	*P* value	Intervention, mean (SD)	Control, mean (SD)	Mean difference, (95% CI)	*P* value
**ASCOT**[a]								
6 months	0.916 (0.096)	0.827 (0.095)	0.089 (0.016, 0.162)	.02	0.866 (0.125)	0.839 (0.124)	0.027 (-0.028, 0.081)	.33
12 months	0.871 (0.097)	0.813 (0.098)	0.058 (-0.013, 0.129)	.11	0.846 (0.124)	0.830 (0.124)	0.017 (-0.041, 0.074)	.56
**EQ-5D-5L**[b]								
6 months	0.777 (0.221)	0.665 (0.218)	0.112 (-0.056 to 0.280)	.18	0.732 (0.213)	0.666 (0.213)	0.066 (-0.027 to 0.159)	.16
12 months	0.643 (0.220)	0.606 (0.221)	0.037 (-0.124 to 0.199)	.64	0.662 (0.212)	0.646 (0.212)	0.016 (-0.082 to 0.114)	.74
**Katz ADL**[c]								
6 months	5.571 (0.745)	5.312 (0.743)	0.259 (-0.309, 0.826)	.36	4.930 (1.068)	4.947 (1.061)	-0.017 (-0.482, 0.448)	.94
12 months	5.319 (0.744)	4.955 (0.746)	0.365 (-0.179, 0.908)	.18	4.743 (1.052)	4.837 (1.052)	-0.094 (-0.583, 0.396)	.70
**IADL**[d]								
6 months	3.050 (0.881)	3.304 (0.876)	-0.254 (-0.923, 0.415)	.44	5.268 (1.088)	4.993 (1.083)	0.276 (-0.198, 0.749)	.25
12 months	3.011 (0.880)	3.259 (0.880)	-0.248 (-0.888, 0.392)	.43	4.930 (1.078)	5.157 (1.075)	-0.226 (-0.723, 0.271)	.37
**GDS**[e]								
6 months	3.095 (1.764)	2.961 (1.760)	0.133 (-1.216, 1.483)	.84	3.680 (1.971)	3.726 (1.960)	-0.045 (-0.904, 0.813)	.92
12 months	3.077 (1.761)	4.109 (1.772)	-1.032 (-2.322, 0.259)	.11	3.972 (1.950)	4.330 (1.948)	-0.358 (-1.257, 0.542)	.43

[a]ASCOT: Adult Social Care Outcomes Toolkit
[b]EQ-5D-5L: EuroQol-5 Dimensions-5
[c]Katz ADL: Katz Index of Independence in Activities of Daily Living.
[d]IADL: Lawton Instrumental Activities of Daily Living Scale.
[e]GDS: Geriatric Depression Scale.

Table S2 Sensitivity analysis for subgroups defined by care package

	CHSP[a]				HCP[b]			
	Intervention, mean (SD)	Control, mean (SD)	Mean difference, (95% CI)	*P* value	Intervention, mean (SD)	Control, mean (SD)	Mean difference, (95% CI)	*P* value
**ASCOT[c]**								
6 months	0.884 (0.107)	0.869 (0.107)	0.015 (-0.044, 0.074)	.61	0.882 (0.123)	0.803 (0.120)	0.079 (0.015, 0.142)	.02
12 months	0.890 (0.107)	0.886 (0.107)	0.004 (-0.063, 0.070)	.91	0.834 (0.123)	0.777 (0.123)	0.057 (-0.005, 0.118)	.07
**EQ-5D-5L[d]**								
6 months	0.850 (0.170)	0.770 (0.170)	0.080 (-0.014 to 0.174)	.09	0.665 (0.237)	0.585 (0.236)	0.080 (-0.043 to 0.204)	.20
12 months	0.755 (0.167)	0.713 (0.167)	0.041 (-0.064 to 0.146)	.43	0.582 (0.237)	0.573 (0.237)	0.009 (-0.109 to 0.128)	.88
**Katz ADL[e]**								
6 months	5.751 (0.611)	5.589 (0.615)	0.162 (-0.176, 0.500)	.34	4.658 (1.168)	4.564 (1.158)	0.094 (-0.518, 0.707)	.76
12 months	5.362 (0.596)	5.457 (0.592)	-0.095 (-0.467, 0.277)	.61	4.601 (1.161)	4.329 (1.167)	0.272 (-0.319, 0.864)	.36
**IADL[f]**								
6 months	5.690 (1.055)	5.757 (1.058)	-0.068 (-0.651, 0.515)	.82	3.786 (1.021)	3.515 (0.996)	0.271 (-0.256, 0.797)	.31
12 months	5.346 (1.047)	5.768 (1.045)	-0.422 (-1.078, 0.234)	.20	3.549 (1.018)	3.776 (1.018)	-0.227 (-0.737, 0.282)	.37
**GDS[g]**								
6 months	3.235 (1.749)	3.108 (1.744)	0.127 (-0.838, 1.092)	.79	3.665 (2.087)	3.998 (2.042)	-0.334 (-1.413, 0.745)	.54
12 months	3.374 (1.738)	3.574 (1.742)	-0.201 (-1.293, 0.892)	.71	3.905 (2.080)	4.975 (2.081)	-1.071 (-2.111, -0.030)	.04

[a]CHSP: Commonwealth Home Support Programme.
[b]HCP: Home Care Package.
[c]ASCOT: Adult Social Care Outcomes Toolkit.
[d]EQ-5D-5L: EuroQol-5 Dimensions-5 Levels.
[e]Katz ADL: Katz Index of Independence in Activities of Daily Living.
[f]IADL: Lawton Instrumental Activities of Daily Living Scale.
[g]GDS: Geriatric Depression Scale.

Table S3 Sensitivity analysis for subgroups defined by living status

	Living alone				Living with others			
	Intervention, mean (SD)	Control, mean (SD)	Mean difference, (95% CI)	*P* value	Intervention, mean (SD)	Control, mean (SD)	Mean difference, (95% CI)	*P* value
**ASCOT[a]**								
6 months	0.874 (0.124)	0.846 (0.124)	0.028 (-0.033, 0.089)	.35	0.883 (0.096)	0.826 (0.095)	0.057 (-0.003, 0.117)	.06
12 months	0.822 (0.123)	0.847 (0.123)	-0.026 (-0.090, 0.039)	.43	0.888 (0.096)	0.796 (0.096)	0.092 (0.036, 0.149)	.002
**EQ-5D-5L[b]**								
6 months	0.803 (0.211)	0.700 (0.211)	0.103 (-0.001 to 0.207)	.05	0.680 (0.212)	0.625 (0.210)	0.055 (-0.076 to 0.187)	.40
12 months	0.591 (0.210)	0.668 (0.210)	-0.077 (-0.187 to 0.033)	.17	0.718 (0.212)	0.596 (0.213)	0.122 (-0.002 to 0.246)	.05
**Katz ADL[c]**								
6 months	5.485 (0.664)	5.244 (0.664)	0.241 (-0.086, 0.567)	.15	4.840 (1.210)	4.697 (1.194)	0.144 (-0.613, 0.900)	.70
12 months	5.058 (0.662)	5.076 (0.662)	-0.018 (-0.365, 0.329)	.92	4.800 (1.202)	4.586 (1.212)	0.214 (-0.509, 0.937)	.55
**IADL[d]**								
6 months	4.979 (0.953)	4.928 (0.957)	0.051 (-0.420, 0.522)	.83	4.275 (1.130)	3.789 (1.121)	0.486 (-0.215, 1.187)	.17
12 months	4.669 (0.952)	4.909 (0.946)	-0.240 (-0.741, 0.261)	.34	3.987 (1.129)	4.152 (1.135)	-0.165 (-0.827, 0.498)	.62
**GDS[e]**								
6 months	3.417 (2.116)	3.333 (2.120)	0.083 (-0.964, 1.131)	.87	3.564 (1.619)	3.751 (1.609)	-0.188 (-1.194, 0.819)	.71
12 months	3.766 (2.101)	4.151 (2.091)	-0.385 (-1.486, 0.717)	.49	3.632 (1.620)	4.356 (1.625)	-0.724 (-1.674, 0.226)	.13

[a]ASCOT: Adult Social Care Outcomes Toolkit.
[b]EQ-5D-5L: EuroQol-5 Dimensions-5 Levels.
[c]Katz ADL: Katz Index of Independence in Activities of Daily Living.
[d]IADL: Lawton Instrumental Activities of Daily Living Scale.
[e]GDS: Geriatric Depression Scale.

Table S4 Baseline characteristics of the participants with and without follow-up data

	6-month follow-up			12-month follow-up		
	Follow-up (n=148)	No Follow-up (n=47)	*P* value	Follow-up (n=130)	No Follow-up (n=65)	*P* value
**ASCOT[a], mean (SD)**	0.846 (0.177)	0.814 (0.163)	.28	0.851 (0.166)	0.812 (0.187)	.14
**EQ5D[b], mean (SD)**	0.709 (0.289)	0.583 (0.318)	.01	0.710 (0.279)	0.615 (0.333)	.04
**Katz ADL[c], mean (SD)**	5.149 (1.411)	4.830 (1.592)	.19	5.115 (1.423)	4.985 (1.536)	.56
**IADL[d], mean (SD)**	5.007 (2.171)	3.809 (2.133)	.001	4.885 (2.264)	4.385 (2.097)	.14
**GDS[e], mean (SD)**	3.095 (2.596)	3.957 (3.270)	.10	3.115 (2.623)	3.677 (3.083)	.19

[a]ASCOT: Adult Social Care Outcomes Toolkit.
[b]EQ-5D-5L: EuroQol-5 Dimensions-5 Levels.
[c]Katz ADL: Katz Index of Independence in Activities of Daily Living.
[d]IADL: Lawton Instrumental Activities of Daily Living Scale.
[e]GDS: Geriatric Depression Scale.
